# Diagnostic Accuracy of Magnetometer-Guided Sentinel Lymphadenectomy After Intraprostatic Injection of Superparamagnetic Iron Oxide Nanoparticles in Intermediate- and High-Risk Prostate Cancer Using the Magnetic Activity of Sentinel Nodes

**DOI:** 10.3389/fphar.2019.01123

**Published:** 2019-10-11

**Authors:** Wiebke Geißen, Svenja Engels, Paula Aust, Jonas Schiffmann, Holger Gerullis, Friedhelm Wawroschek, Alexander Winter

**Affiliations:** University Hospital for Urology, Klinikum Oldenburg, School of Medicine and Health Sciences, Carl von Ossietzky University Oldenburg, Oldenburg, Germany

**Keywords:** superparamagnetic iron oxide nanoparticle, prostate cancer, sentinel node, lymphadenectomy, magnetometer, lymph node metastases, nomogram

## Abstract

Due to the high morbidity of extended lymph node dissection (eLND) and the low detection rate of limited lymph node dissection (LND), targeted sentinel lymph node dissection (sLND) was implemented in prostate cancer (PCa). Subsequently, nonradioactive sentinel lymph node (SLN) detection using magnetic resonance imaging (MRI) and a magnetometer after intraprostatic injection of superparamagnetic iron oxide nanoparticles (SPIONs) was successfully applied in PCa. To validate the reliability of this approach, considering the magnetic activity of SLNs or whether it is sufficient to dissect only the most active SLNs as shown in other tumor entities for radio-guided sLND, we analyzed magnetometer-guided sLND results in 218 high- and intermediate-risk PCa patients undergoing eLND as a reference standard. Using a sentinel nomogram to predict lymph node invasion (LNI), a risk range was determined up to which LND could be dispensed with or sLND only would be adequate. In total, 3,711 LNs were dissected, and 1,779 SLNs (median, 8) were identified. Among 78 LN-positive patients, there were 264 LN metastases (median, 2). sLND had a 96.79% diagnostic rate, 88.16% sensitivity, 98.59% specificity, 97.1% positive predictive value (PPV), 93.96% negative predictive value (NPV), 4.13% false-negative rate, and 0.92% additional diagnostic value (LN metastases only outside the eLND template). For intermediate-risk patients only, the sensitivity, specificity, PPV, and NPV were 100%. Magnetic activities of SLNs were heterogeneous regardless of metastasis. The accuracy of predicting the presence of metastases for each LN from the proportion of activity was only 57.3% in high- and 65% in intermediate-risk patients. Patients with LNI risk of less than 5% could have been spared LND, as no positive LNs were found in this group. For patients with an LNI risk between 5% and 20%, sLND-only would have been sufficient to detect almost all LN metastases; thus, eLND could be dispensed with in 36% of patients. In conclusion, SPION-guided sLND is a reliable alternative to eLND in intermediate-/high-risk PCa. No conclusions can be drawn from magnetic SLN activity regarding the presence of metastases. LND could be dispensed with according to a nomogram of predicted probability for LNI of 5% without losing any LN-positive patient. Patients with LNI risk between 5% and 20% could be spared eLND by performing sLND.

## Introduction

Lymph node (LN) status plays a decisive role in estimating the local extent and prognosis of prostate cancer (PCa), as survival is inversely related to the number of positive nodes (Masterson et al., 2006). Patients classified as intermediate- or high-risk based on clinical tumor category, biopsy Gleason score, and pretreatment prostate-specific antigen (PSA) levels have a significantly elevated risk of experiencing metastatic progression compared with low-risk PCa. Therapeutic options include radical prostatectomy (RP) combined with pelvic LN dissection (LND) if the risk of LN invasion (LNI) is greater than 5% or local radiotherapy (Mottet et al., 2017). Besides LND being the most reliable procedure for LN staging for patients with clinically localized PCa, it is also important for planning appropriate adjuvant therapy (Withrow et al., 2011; Mottet et al., 2017). Furthermore, there is increasing evidence of positive therapeutic effects of pelvic LND or resecting LN metastases, particularly in patients with minimal LNI (Allaf et al., 2004; Withrow et al., 2011; Seiler et al., 2014; Winter et al., 2015a).

Growing evidence in the setting of recurrent PCa argues for the use of prostate-specific membrane antigen positron emission tomography–computed tomography (PSMA PET/CT) for detection of LN metastasis in men with persistent PSA after RP. PSMA PET/CT imaging at primary staging might improve the detection of LN metastases compared to conventional imaging techniques, too. However, there is still a paucity of data comparing PSMA PET/CT results from primary staging with histological results from pelvic LND. Only few reports involving few patients exist pursuing this purpose. Some of these studies could show a high sensitivity in the detection of LN metastases (Cantiello et al., 2017). In principle, the reliability of these procedures is limited by their spatial resolution, which limits the sensitivity of detecting LN (micro)metastases (<2 mm). In early stages of metastasis, the metastatic cells reside in the hypoxic regions of the subcapsular sinus of the LN. At this initial unobtrusive stage in disease progression, the single cells or micrometastases have not yet connected to the blood circulation. Thus, targeting is likely to be possible only *via* the lymphatic network using imaging agents or sentinel tracers that exhibit a lymphatic drainage from the primary tumor location (van Leeuwen et al., 2019).

LND can be performed as extended or limited procedures to various extents. Several studies have shown that extended LND (eLND) including the area along the common, external, and internal iliac vessels as well as the obturator fossa region has a more reliable diagnostic value and could improve biochemical recurrence-free survival compared with the limited approach (Allaf et al., 2004; Briganti et al., 2009a; Abdollah et al., 2012; Bivalacqua et al., 2013). Although the extent of LND correlates with improved staging accuracy, it also correlates with a higher morbidity, such as an increased risk of lymphoceles, thromboembolism, and intraoperative injury of contiguous anatomical structures. Thus, it is of special interest to find a compromise between high diagnostic sensitivity and low risk of perioperative complications (Briganti et al., 2006a; Briganti et al., 2009a; Winter et al., 2011; Bivalacqua et al., 2013). Winter et al. (2011) could show that pelvic sentinel LN (SLN) dissection (sLND) using a radioactive tracer has, despite the dissection of LNs in difficultly accessible regions (presacral, iliaca interna region), a lower complication rate than the extended lymphadenectomy approach. In addition to decreasing the severity of surgical intervention, fewer LNs also enable more precise histopathological examination, so that even micrometastases are more likely found.

In 1960, Gould et al. (1960) were the first to describe the primary draining LN as SLN in their studies of parotid carcinoma. In 1977, Cabanas (1977) described the lymph pathway of the penis and found that the SLN was often the only positive LN in penis carcinoma, so a more extensive lymphadenectomy may not be necessary. sLND has become an established procedure in tumor diagnostics and therapy of certain tumor types with the aim of reducing the number of LNs being removed and thus the complication rate, while maintaining a high sensitivity for metastasis detection through targeted removal. To improve LND in PCa, Wawroschek et al. (1999) transferred the sentinel technique from other tumor entities such as breast cancer and malignant melanoma to PCa (Cabanas, 1977; Morton et al., 1992; Giuliano et al., 1994; Wawroschek et al., 1999). Their first results suggested the high sensitivity of sLND for metastasis detection could significantly minimize the extent and duration of surgery. Wit et al. (2017) demonstrated the high diagnostic reliability of sLND comparable with eLND in a systematic review of 21 studies, which is reflected in an estimated overall median sensitivity of 95.2% and specificity of 100%. Holl et al. (2009) demonstrated that sLND was a reliable addition or even alternative to eLND in PCa using ^99m^technetium (^99m^Tc) nanocolloid as a tracer. However, the radioactive tracer is accompanied by several disadvantages, particularly the dependence on radioisotopes and exposure to ionizing radiation. Therefore, SLN detection using a magnetic tracer was established in breast cancer, as equivalent results to ^99m^Tc nanocolloid could be shown (Douek et al., 2014). Winter et al. (2014) were the first to use intraprostatic injection of superparamagnetic iron oxide nanoparticles (SPIONs) to detect SLNs in PCa. Initial results including 19 patients were convincing, showing a detection rate of 90%, whereas the first examinations of breast cancer were only at 77% (Winter et al., 2014). Later studies resulted in a diagnostic rate of 100% (Winter et al., 2017a). Winter et al. (2017a; 2018a) also demonstrated an additional diagnostic value through detecting SLNs outside the established eLND template. Another advantage is that SLNs can be visualized preoperatively using magnetic resonance imaging (MRI) after intraprostatic injection of SPIONs, allowing a higher spatial resolution than lymphoscintigraphy (Winter et al., 2018a; Winter et al., 2018b).

In 2000, Martin et al. (2000) were the first to introduce the “10% rule” for breast cancer using both blue dye and radioactive colloid injection. In their prospective multicenter study, they concluded that all blue nodes and all nodes with 10% or more of the count of the hottest SLNs should be harvested for optimal nodal staging. Since then, several other groups have verified this method for breast cancer and malignant melanoma to reduce the number of dissected LNs without a loss of reliability in identifying LN-positive patients (Carlson et al., 2002; Chung et al., 2008). In both tumor entities, the sLND under consideration of the 10% rule was mostly seen as a good alternative to the eLND.

In this study we examined, for the first time, whether it might also be sufficient to dissect only the most active LNs in PCa. In this retrospective study, a larger collective than in previous studies was included to determine the diagnostic accuracy of SPION-based sLND compared with conventional eLND. We aimed to investigate the influence of tracer uptake or the level of magnetic activity in SLNs on the detection of LN metastases in PCa. A risk stratification of all patients with lymphogenic metastasis on the basis of a sentinel-based nomogram developed by Winter et al. (2017b) to predict the probability of LN metastases was performed. Probabilities were compared to results of sLND, and a threshold was determined up to which an LND can be completely dispensed with or sLND would be sufficient.

## Materials and Methods

### Study Design

This retrospective study included a prospective documented cohort of 218 patients with intermediate- or high-risk PCa who underwent open radical retropubic prostatectomy with a magnetometer-guided sLND followed by an eLND. All procedures were performed at the University Hospital for Urology Oldenburg between February 24, 2015, and October 23, 2018.

Inclusion criteria were at least 18 years in age, intermediate- or high-risk PCa using the European Association of Urology classification (intermediate-risk: PSA 10–20 ng/ml and/or Gleason score = 7 and/or cT2b; high-risk: PSA >20 ng/ml and/or Gleason score >7 and/or cT2c), and receiving the surgical intervention named above (Mottet et al., 2017).

Exclusion criteria were patients with previous transurethral or open prostate surgeries, previous hormone therapies, known intolerance or hypersensitivity to iron, dextran compounds or Sienna+^®^, iron overload disease, and those with pacemakers or other implantable devices in the chest wall as well as hip prosthesis or other metallic pelvic implant.

### Magnetic SPION Tracer and Magnetometer

The SentiMag^®^ system (Endomagnetics Ltd., Cambridge, UK) was applied to mark and detect LNs in cancer patients and is composed of a handheld magnetometer, the SentiMag^®^ unit, and the Sienna+^®^ magnetic tracer, which both are Conformité Européenne (CE) certified as class IIa medical devices.

Sienna+^®^ is a suspension of superparamagnetic carboxydextran-coated iron oxide particles in injectable water. Each milliliter of Sienna+^®^ contains approximately 28 mg of iron. The mean hydrodynamic diameter of the particles is 60 nm, just as ^99m^Tc nanocolloid, but more homogeneous. Their functional properties are also comparable, as after interstitial injection both flow through the lymph system and get trapped in SLNs where Sienna+^®^ subsequently forms iron deposits that can be intraoperatively identified with the handheld SentiMag^®^ magnetometer. Another advantage of Sienna+^®^ is that its brown color can also help identify SLNs.

Regarding nonclinical toxicology, Sienna+^®^ has been reviewed and tested as specified in EN 10993-1:2009 based on the specified site of injection and duration. Sienna+^®^ is contraindicated in any patient with hypersensitivity to iron oxide or dextran compounds and should not be administered in any patient with an iron overload disease or with a metal implant close to the expected SLN location. Sienna+^®^ is only approved for interstitial injection. When similar material to that used in Sienna+^®^ has been injected intravenously, the following undesirable effects have been reported: common (<2%)—pain at the injection site, vasodilation, paresthesia; uncommon (≥0.1% to <1%)—asthenia, back pain, injection site reactions, chest pain, nausea, vomiting, headache, taste perversion, pruritus, and rash; rare (≥0.01% to <0.1%)—hypersensitivity and anaphylaxis, hypertension, phlebitis, hyperesthesia, anxiety, dizziness, convulsion, parosmia, dyspnea, increased cough, rhinitis, eczema, and urticaria. There have been a small number of reports of inflammatory and hypersensitivity response with intradermal injection. There is no evidence of adverse reaction following interstitial injection (Endomagnetics, 2015).

### Tracer Injection

A special feature of PCa is that it is mostly a multifocal disease, which does not allow reliable localization of the index lesion. Thus, it is not possible to simply inject the tracer in peritumoral regions to observe lymphatic drainage only of the tumor, as it is done in other tumor entities such as breast cancer and malignant melanoma. Therefore, it is particularly important to image every primary draining LN of the entire prostate, so that the SLN of the cancer will definitely be included.

In this study, all patients received an ultrasound-guided transrectal injection of 2 ml Sienna+^®^ into the prostate the day before surgery. Based on our experiences with various injection techniques and the results of others, one urologist injected 1 ml in each side, which were evenly spread as three peripheral deposits (Wawroschek et al., 2003; Buckle et al., 2012).

### SPION-MRI

Most patients received preoperative MRI after the SPION injection to visualize SLNs to help the surgeon find them intraoperatively. As previously described, the possibility of radioactive-free visualization with MRI offers a decisive advantage over ^99m^Tc nanocolloid-based lymphoscintigraphy (McCauley et al., 2002; Iida et al., 2013; Pouw et al., 2015; Winter et al., 2018a; Winter et al., 2018b).

### Surgical Procedure and Histopathological Examination

One day after tracer injection, all patients underwent open magnetometer-guided (SentiMag^®^) sLND and eLND as a reference, followed by a radical retropubic prostatectomy performed by four high-volume surgeons.

Using the handheld magnetometer (SentiMag^®^), magnetic activity was detected, and the corresponding lymphatic fatty tissue containing the active LN and if applicable direct adherent nonactive LNs was dissected. To generate values, the magnetometer measures the difference between the local magnetic activity and the activity in the surrounding medium. For this purpose, it is necessary to calibrate the magnetometer against the corresponding medium before measuring. The measurand is “units” and thus is not defined. To not disturb the magnetic field of the SentiMag^®^ system, metal retractors were replaced with polymer retractors (SUSI^®^, Aesculap^®^; B. Braun Melsungen AG, Melsungen, Germany).

Thereafter, eLND was conducted to remove remaining lymphatic fatty tissue from the eLND template including the area along the common, external, and internal iliac vessels, as well as the obturator fossa region.

Postoperatively, prostatectomy specimens and dissected LNs were routinely processed. All LNs were cut in 3-mm transverse sections and embedded in paraffin. Then 4- to 5-μm-thick sections were cut and stained with hematoxylin-eosin. For individual cases in which conventional histology did not yield clear results, immunohistochemistry with a pancytokeratin antibody (AE1/AE3) was performed to assess metastatic spread.

### Data Analysis

In this study, we defined a SLN as an *in vivo* magnetically active LN. Based on previous work, eLND was used as a reference standard to assess the diagnostic reliability of sLND as the index test (van der Poel et al., 2017; Winter et al., 2017a; Wit et al., 2017). Data were summarized in a 2 × 2 cross table to calculate the diagnostic rate (number of patients with detected SLNs/total number operated), sensitivity, specificity, positive predictive value (PPV), negative predictive value (NPV), false-negative (FN) rate, and false-positive (FP) rate in both risk subgroups, with FN and FP rates being defined as established in previous work (Wit et al., 2017). Patients were classified as FN if all SLNs were histologically negative, but metastasis was found in inactive LNs in the eLND template. Patients were classified as FP if SLNs outside the eLND template were histologically positive, while no metastases were detected in the eLND template. Consequently, a high FP rate provides a diagnostic benefit of sLND over eLND and reflects the inadequacy of eLND as a staging procedure.

In addition to patient-based evaluation, a node-based analysis was performed to determine the FN rate of sLND. In this case, an LN was defined as FN if it showed no magnetic activity despite the presence of a metastasis. To determine whether it is sufficient to dissect only the most active SLNs in PCa and still correctly identify LN-positive patients, the activity of metastasis-positive LNs was examined in patients with LN metastases. Predictive accuracy was quantified using the receiver operating characteristic (ROC) curve.

The updated nomogram developed by Winter et al. (2017b) (2017) based on preoperative PSA, clinical T category, primary and secondary Gleason grade, and the percentage of positive cores was used for risk stratification to predict the probability of LN metastases. Statistical analyses were performed using SPSS Statistics 25 (IBM, Armonk, NY, USA).

### Ethical Approval

All subjects gave informed consent for the documentation and scientific evaluation of their data in a prospective clinical registry. The study was conducted in accordance with the Declaration of Helsinki, and the protocol was registered in an international clinical trial register (Research Registry: researchregistry4963). The study protocol was approved by the Medical Ethics Committee of the Carl von Ossietzky University Oldenburg (no. 2018-140).

## Results

### Diagnostic Accuracy of sLND

This study included 218 intermediate- (n = 97) and high-risk (n = 121) PCa patients who underwent magnetometer-guided sLND, eLND, and RP after intraprostatic SPION injection one day before surgery. **Table 1** summarizes patient characteristics. SPION-tracer injections proceeded without any complications. None of the 218 patients exhibited adverse events attributable to SPION injection.

**Table 1:** Patient characteristics.

	Overall n = 218 (100%)	Patients with high-risk PCa n = 121 (55.5%)	Patients with intermediate-risk PCa n = 97 (44.5%)
Age, y (median) IQR	69 64–73	69 64–73	68 63–73
Total PSA, ng/ml (median) IQR	10.95 6.89–20.19	15.40 8.27–30.33	8.18 5.82–12.35
Tumor stage (%)			
T1c	94 (43.1)	24 (19.8)	70 (72.2)
T2a	18 (8.3)	8 (6.6)	10 (10.3)
T2b	24 (11.0)	7 (5.8)	17 (17.5)
T2c	62 (28.4)	62 (51.2)	0
T3a	16 (7.3)	16 (13.2)	0
T3b	4 (1.8)	4 (3.3)	0
Biopsy Gleason score (%) 6 (3 + 3) 7 (3 + 4) 7 (4 + 3) 8 (4 + 4) 8 (3 + 5) 8 (5 + 3) 9 (4 + 5) 9 (5 + 4) 10 (5 + 5)	18 (8.3) 115 (52.8) 29 (13.3) 38 (17.4) 1 (0.5) 1 (0.5) 12 (5.5) 3 (1.4) 1 (0.5)	8 (6.6) 42 (34.7) 15 (12.4) 38 (31.4) 1 (0.8) 1 (0.8) 12 (9.9) 3 (2.5) 1 (0.8)	10 (10.3) 73 (75.3) 14 (14.4) 0 0 0 0 0 0
Postoperative Gleason score (%) 6 (3 + 3) 7 (3 + 4) 7 (4 + 3) 8 (4 + 4) 9 (4 + 5) 9 (5 + 4)	3 (1.4) 85 (39.0) 65 (29.8) 35 (16.1) 29 (13.3) 1 (0.5)	1 (0.8) 27 (22.3) 39 (32.2) 27 (22.3) 26 (21.5) 1 (0.8)	2 (2.1) 58 (59.8) 26 (26.8) 8 (8.2) 3 (3.1) 0
Pathologic stage (%)			
pT2a	4 (1.8)	0	4 (4.1)
pT2c	86 (39.4)	30 (24.8)	56 (57.7)
pT3a	42 (19.3)	22 (18.2)	20 (20.6)
pT3b	83 (38.1)	66 (54.5)	17 (17.5)
pT4	3 (1.4)	3 (2.5)	0

Data are given as median (IQR) or number (%). IQR, interquartile range; PSA, prostate-specific antigen.

In total, 3,711 LNs were dissected and 1,779 SLNs were found in 211 of 218 patients (diagnostic rate of 96.79%), with a median of eight SLNs. Seventy-eight patients (29.55%) were LN-positive and had 264 LN metastases (median = 2). A LN-positive patient was correctly identified by sLND with an 88.16% sensitivity, 98.59% specificity, 97.1% PPV, and 93.96% NPV (**Table 2**). sLND did not detect nine LN-positive patients who were detected by eLND, resulting in a FN rate of 4.13%. However, sLND showed additional diagnostic value or detected LN metastases in two patients outside the eLND template, where eLND did not reveal any metastases (0.92% FP rate). Considering every positive LN for itself with the aim of not missing any, sLND had a 48.48% FN rate. There were 128 of 264 positive LNs that did not show magnetic activity.

**Table 2:** 2 × 2 Table to calculate diagnostic test accuracy of magnetometer-guided sentinel lymph node dissection in the overall cohort including intermediate- and high-risk prostate cancer patients (n = 218).

		LN status according to reference standard (eLND)		
		LN +	LN −	Total
**Index test**	**SLN +**	67	2	69
	**SLN −**	9	140	149
	**Total**	76	142	218

Extended pelvic lymphadenectomy was used as a reference standard to calculate diagnostic accuracy of magnetometer-guided sentinel lymph node dissection for detecting lymph node–positive patients in the overall cohort.

LN, lymph node; eLND, extended lymph node dissection; SLN, sentinel lymph node.

In the high-risk group (n = 121), a total of 2,074 LNs were dissected. Altogether, 967 SLNs were detected in 116 of 121 patients (95.87% diagnostic rate), with a median of eight SLNs per patient. There were 238 LN metastases (median = 2) found in 65 patients (53.72%). Patient-based sLND results had an 85.71% sensitivity, 96.55% specificity, 96.43% PPV, and 86.15% NPV (**Table 3**). sLND detected two patients with LN metastases outside the eLND template, where eLND did not reveal any metastases (1.65% FP rate), meaning additional diagnostic value could be shown. Nine LN-positive patients could not be detected (7.44% FN rate); four patients did not have any active LNs, two had one SLN, and one patient had two SLNs. In two of the SLN-negative patients, the tracer was not correctly distributed intraprostatically or was mostly found in the perirectal region, probably due to advanced PCa or insufficient injection. LN–based sLND had a 50.84% FN rate, as 121 of 238 positive LNs did not show magnetic activity.

**Table 3:** 2 × 2 Table to calculate diagnostic test accuracy of magnetometer-guided sentinel lymph node dissection in high-risk prostate cancer patients (n = 121).

		LN status according to reference standard (eLND)		
		LN +	LN −	Total
**Index test**	**SLN +**	54	2	56
	**SLN −**	9	56	65
	**Total**	63	58	121

Extended pelvic lymphadenectomy was used as a reference standard to calculate diagnostic test accuracy of magnetometer-guided sentinel lymph node dissection for detecting lymph node–positive patients among high-risk prostate cancer patients.

LN, lymph node; eLND, extended lymph node dissection; SLN, sentinel lymph node.

An even higher diagnostic reliability result was found for the intermediate-risk group. A total of 1,637 LNs were dissected in this risk group, and 812 SLNs were found in 95 of 97 patients, yielding a diagnostic rate of 97.94%, with a median of eight SLNs. Among the intermediate-risk patients, 13.4% (n = 13) were LN-positive and had 26 LN metastases (median = 1). All of them also had positive SLNs. Patient-based results showed 100% sensitivity, 100% specificity, 100% PPV, and 100% NPV (**Table 4**). None of the LN-positive patients were missed (0% FN rate), and the FP rate was 0%. If we consider every positive LN for itself, seven of 26 positive LNs were magnetically inactive, although they were histopathologically positive for metastasis (26.92% FN rate).

**Table 4:** 2 × 2 Table to calculate diagnostic test accuracy of magnetometer-guided sentinel lymph node dissection in intermediate-risk prostate cancer patients (n = 97).

		LN status according to reference standard (eLND)		
		LN +	LN −	Total
**Index test**	**SLN +**	13	0	13
	**SLN −**	0	84	84
	**Total**	13	84	97

Extended pelvic lymphadenectomy was used as a reference standard to calculate diagnostic test accuracy of magnetometer-guided sentinel lymph node dissection for detecting lymph node–positive patients among intermediate-risk prostate cancer patients.

LN, lymph node; eLND, extended lymph node dissection; SLN, sentinel lymph node.

### Magnetic Activity

**Figure 1:**
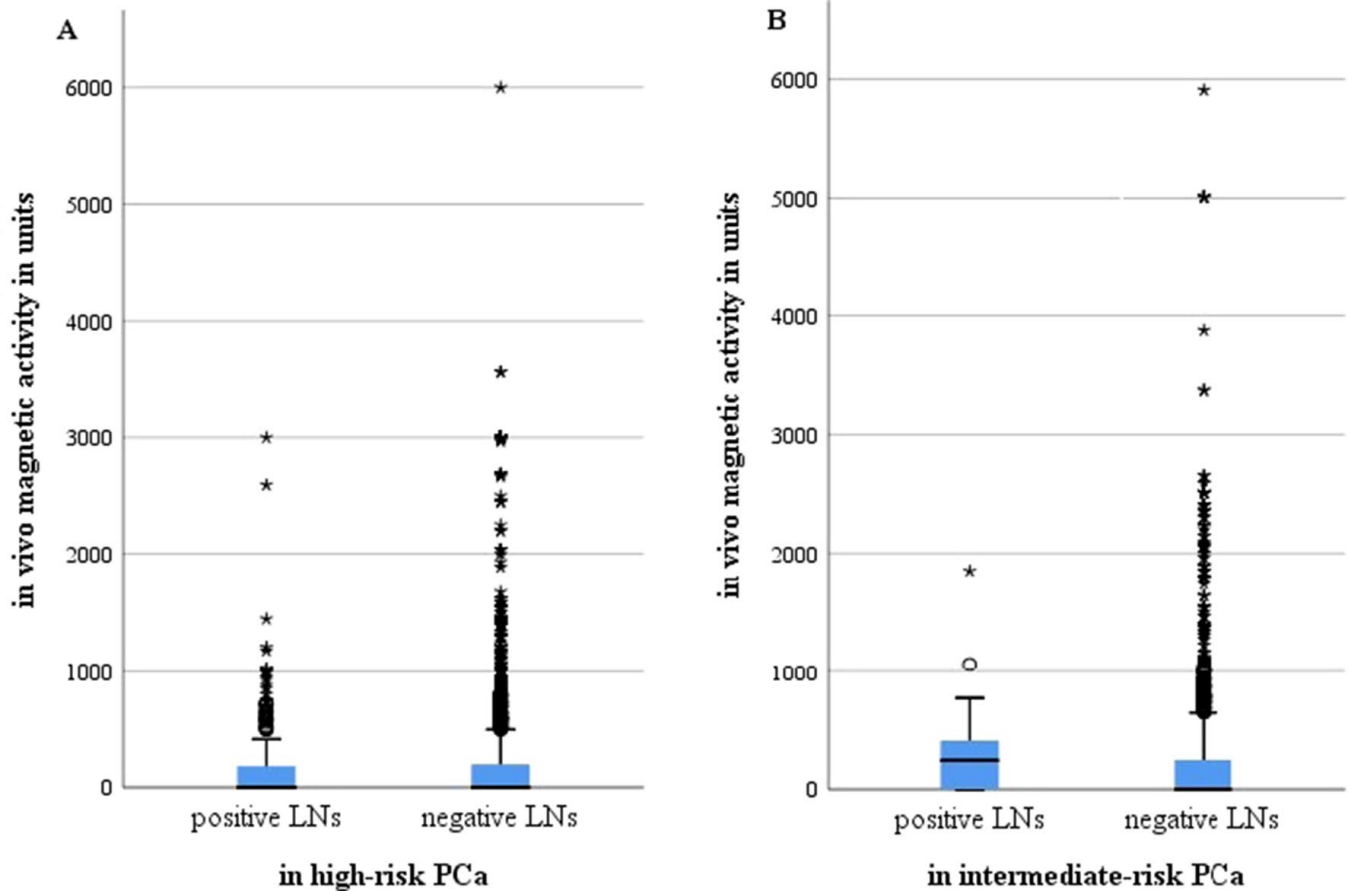
Magnetic activity in units for all metastasis positive and negative lymph nodes after intraprostatic injection of SPIONs in intermediate- and high-risk prostate cancer patients (n = 218). **(A)** High-risk prostate cancer patients, n = 121; **(B)** intermediate-risk prostate cancer patients, n = 97. LNs, lymph nodes; PCa, prostate cancer.°, minor outliers, a point that falls outside the data set's inner fences;*, major outliers, a point that falls outside the outer fences.

Examining correlations between levels of *in vivo* SLN magnetic activity and existing metastases, a mean activity of positive as well as of negative LNs in both risk groups between 184 and 323 units was revealed with a maximum of 6,000 units (**Figure 1**). Although the positive LNs in intermediate-risk patients showed the highest mean activity (323 units), the 95% confidence interval (156–491) also included the mean activity of negative LNs (273 units). The median for positive and negative LNs in high-risk patients was 0, likewise for negative LNs in intermediate-risk patients. Positive LNs in the intermediate-risk group had a median magnetic activity of 249.5.

**Figure 2:**
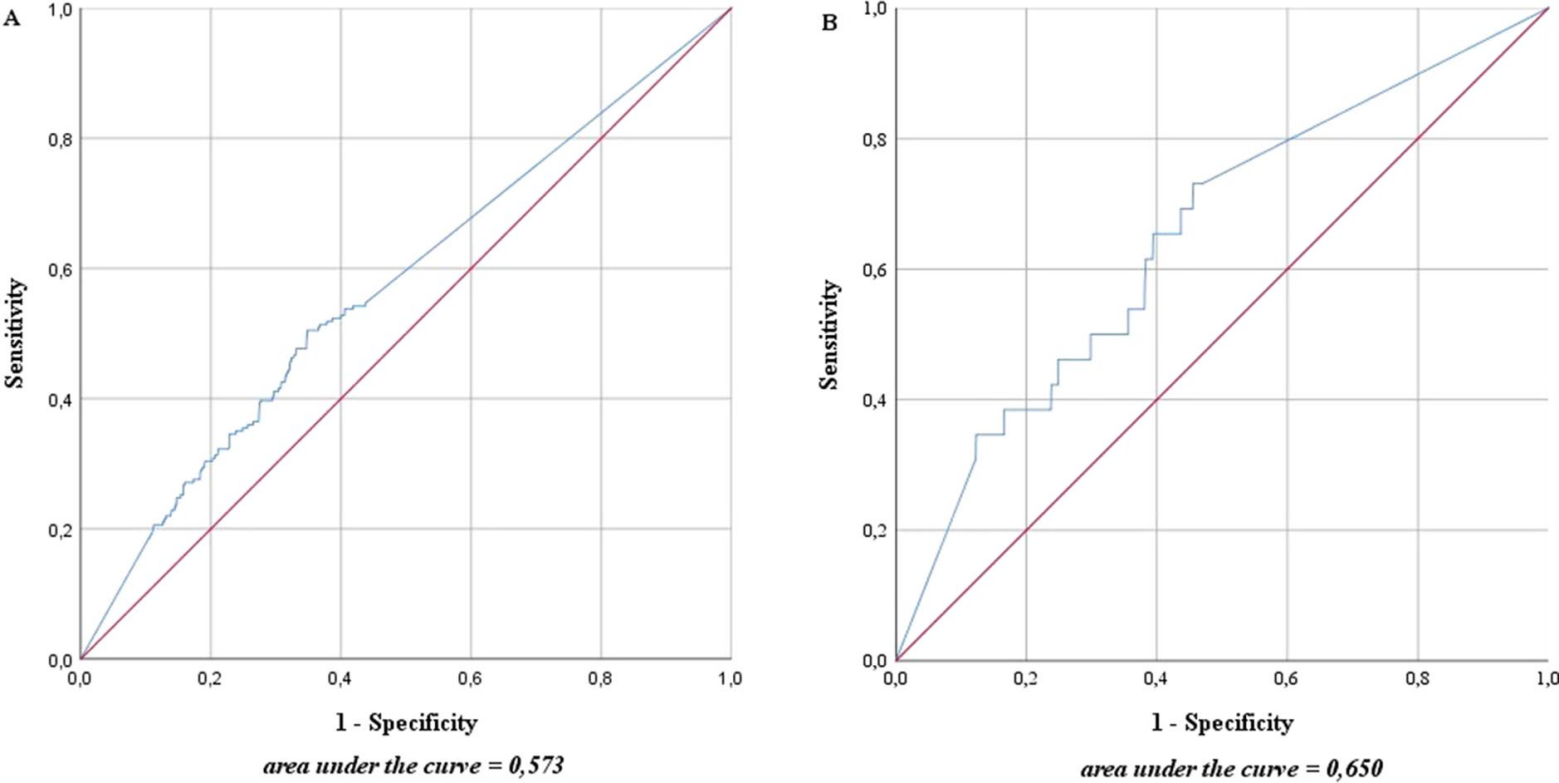
Accuracy with which one can deduce the presence of metastases for each lymph node from the proportion of magnetic activity compared to the measured maximum of the same patient in intermediate- and high-risk prostate cancer patients (n = 218). **(A)** High-risk prostate cancer patients, n = 121; **(B)** intermediate-risk prostate cancer patients, n = 97. The blue line represents the prediction accuracy of the presence of metastases based on magnetic activity. The red line is a reference for prediction accuracy that corresponds to randomness.

The ROC curve in **Figure 2** shows the accuracy with which one can deduce the presence of metastases for each LN from the proportion of activity compared with the measured maximum of the same patient. The area under the curve (AUC) was as low in high-risk as in intermediate-risk patients (57.3% and 65.0%, respectively).

**Figure 3:**
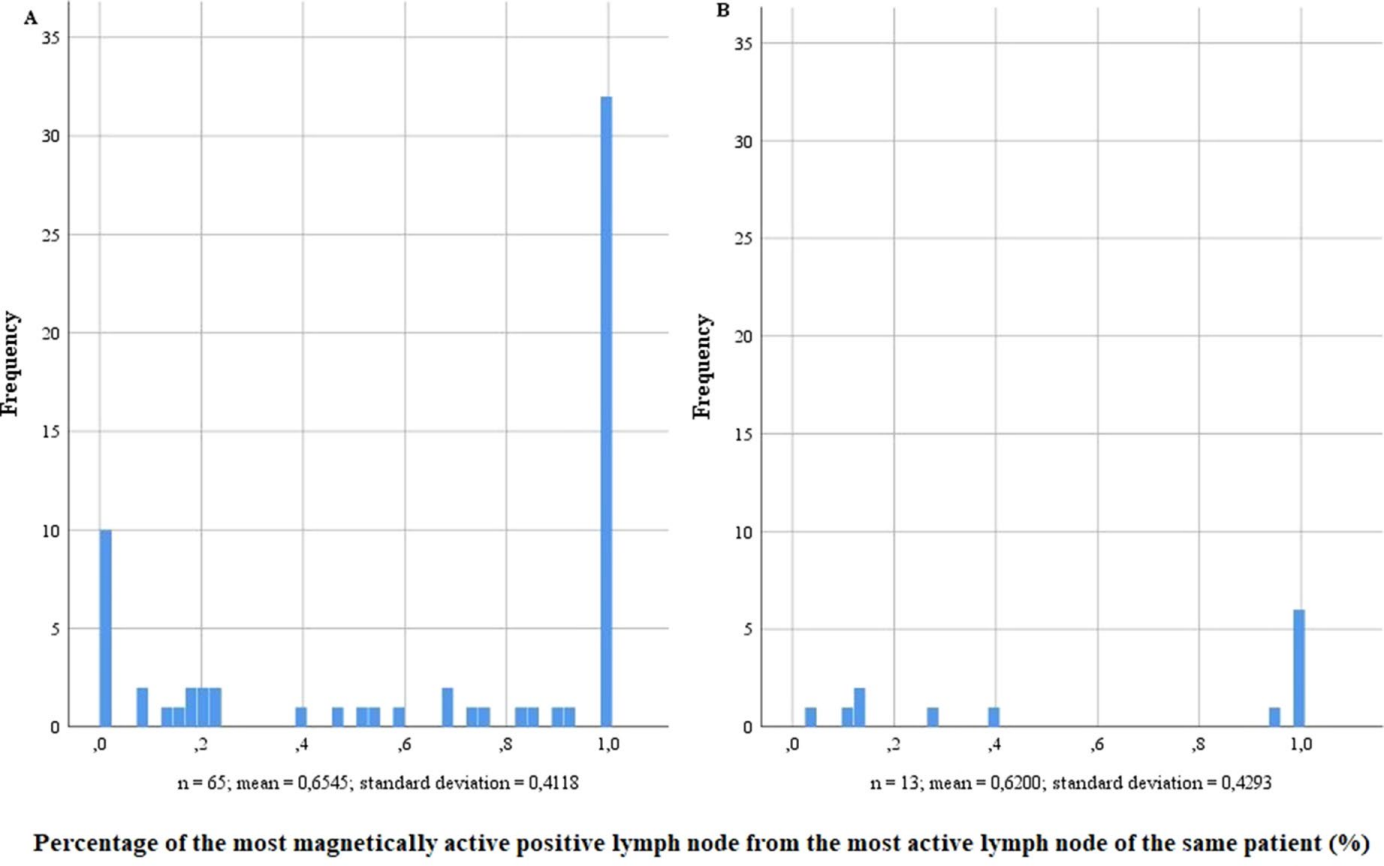
Percentage of the most magnetically active positive lymph node from the most active lymph node of the same patient in lymph node–positive intermediate- and high-risk prostate cancer patients (n = 218). **(A)** High-risk prostate cancer patients, n = 121; **(B)** intermediate-risk prostate cancer patients, n = 97.

Conspicuously, in both risk groups, most LN-positive patients also showed metastases in the SLN with the highest activity (**Figure 3**). However, LN-positive patients without metastases in the most active SLN also existed. Especially in high-risk patients, nine of the 65 LN-positive patients did not have a positive LN with magnetic activity, while four did not have any active LN. In the intermediate-risk group, every LN-positive patient had at least one positive LN with magnetic activity. When comparing the most active positive LN with the highest magnetic activity of the same patient, no matter if the highest activity was found in a positive or negative LN, the percentages in the intermediate-risk collective were between 3.95% and 100%.

### Nomogram-Based Risk Stratification

**Figure 4:**
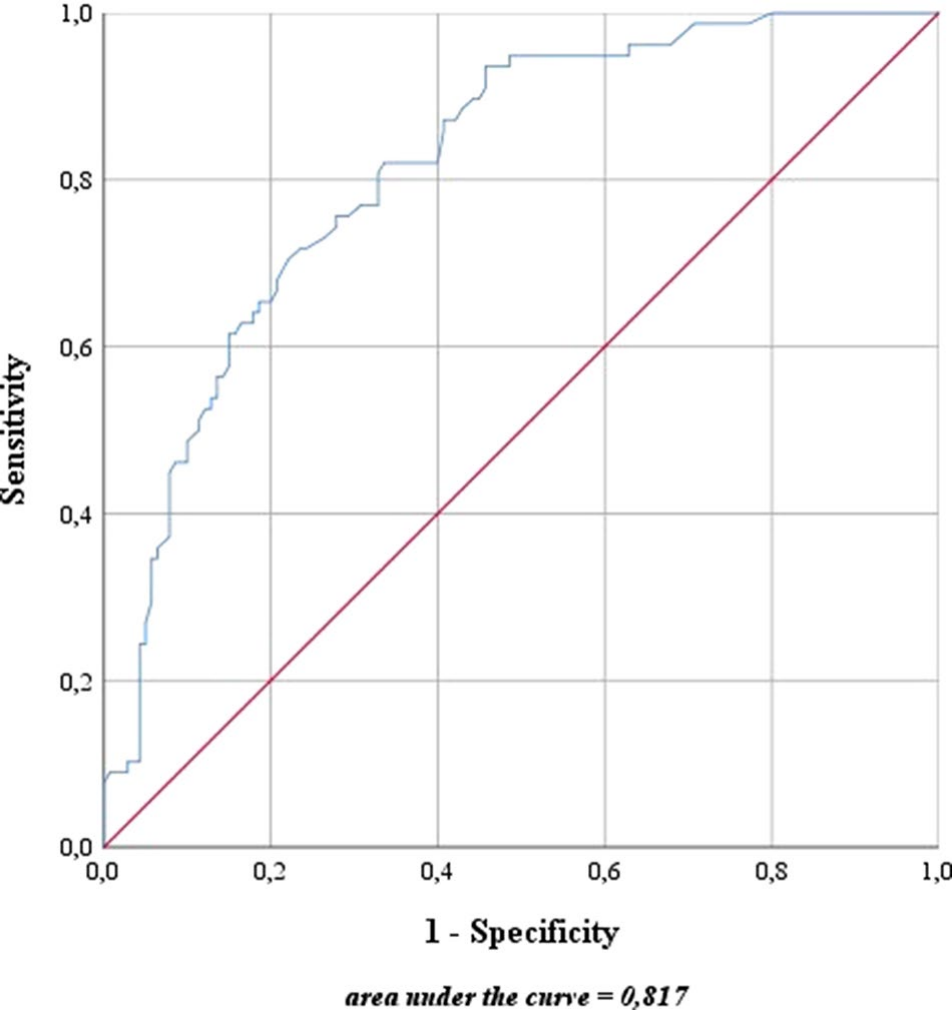
Predictive accuracy for the presence of lymph node invasion based on the updated nomogram of Winter et al. (2017b). The blue line represents the predictive accuracy for lymph node invasion in this cohort based on the nomogram of Winter et al. (2017b). The red line is a reference for a predictive accuracy that corresponds to randomness.

For risk stratification using the updated sentinel nomogram created by Winter et al. (Winter et al., 2017b) in 2017 based on preoperative PSA, clinical T category, primary and secondary Gleason grade, and the percentage of positive cores, high- and intermediate-risk patients were merged to one group. The nomogram was used to categorize the probability of LNI for each patient. The overall predictive accuracy (AUC) for the probability of LNI was 81.7% in this cohort (**Figure 4**). Furthermore, the nomogram was used to determine a cutoff value aimed at removing all positive LNs in view of a possible therapeutic benefit in addition to the diagnostic value of LND.

None of the 11 patients with a risk of lymphogenic metastasis of ≤5% had positive LNs. Twenty-seven of the 130 patients with a risk of LNI between 5.01% and 45% were identified as positive by eLND. All of them were already detected in the preceding sLND. Nine of the 77 patients in the risk range 45.01% to 100% had metastases in non-SLNs that were only detected by performing additional eLND. However, two patients who only had metastases outside the eLND template were found by sLND (**Tables 5** and **6**). In node-based analysis of nine patients with an LNI risk of 5.01% to 20%, all positive LNs 14 out of 15 were detected using sLND. In the risk range of 20.01% to 100%, positive LNs were detected in both sLND and eLND, but with none of these LND methods alone could all positive LNs be identified (**Tables 7** and **8**).

**Table 5:** Table to calculate the predictive accuracy of detecting lymph node–positive patients based on the updated nomogram of Winter et al. (2017b) using sentinel lymph node dissection as a reference standard.

Nomogram-based risk group (%)	sLND right-positive patients (n = 69)	Patients without histologic LNI and negative SLNs; "right-negative" (n = 140)	Patients with histologic LNI and negative SLNs; "false-negative" (n = 9)	Patients with histological LNI and positive SLNs; "right-positive" (n = 218)
0–5	0	11	0	11
5.01–10	3	40	0	43
10.01–15	1	15	0	16
15.01–20	5	14	0	19
20.01–25	5	7	0	12
25.01–30	4	10	0	14
30.01–35	2	4	0	6
35.01–40	2	5	0	7
40.01–45	5	8	0	13
45.01–50	6	7	1	14
50.01–55	0	0	2	2
55.01–60	4	5	0	9
60.01–65	7	3	0	10
65.01–70	3	3	1	7
70.01–75	6	1	0	7
75.01–80	1	1	2	4
80.01–85	9	0	1	10
85.01–90	1	3	0	4
90.01–95	2	3	0	5
95.01–100	3	0	2	5

The nomogram-based risk groups were calculated by applying the updated nomogram of Winter et al. (2017b). SLN, sentinel lymph node; LNI, lymph node invasion.

**Table 6:** Table to calculate the predictive accuracy of detecting lymph node–positive patients based on the updated nomogram of Winter et al. (2017b) using extended lymph node dissection as a reference standard.

Nomogram- based risk group (%)	Patients with histological LNI and positive LNs detected by eLND; "right-positive" (n = 76)	Patients without histological LNI and negative LNs detected by eLND; "right-negative" (n = 140)	Patients with histological LNI and without positive LNs detected by eLND; "false-negative" (n = 2)	Total (n = 218)
0–5	0	11	0	11
5.01–10	3	40	0	43
10.01–15	1	15	0	16
15.01–20	5	14	0	19
20.01–25	5	7	0	12
25.01–30	4	10	0	14
30.01–35	2	4	0	6
35.01–40	2	5	0	7
40.01–45	5	8	0	13
45.01–50	7	7	0	14
50.01–55	2	0	0	2
55.01–60	4	5	0	9
60.01–65	6	3	1	10
65.01–70	4	3	0	7
70.01–75	6	1	0	7
75.01–80	3	1	0	4
80.01–85	9	0	1	10
85.01–90	1	3	0	4
90.01–95	2	3	0	5
95.01–100	5	0	0	5

The nomogram-based risk groups were calculated by applying the updated nomogram of Winter et al. (2017b). eLND, extended lymph node dissection. sLND, sentinel lymph node dissection; SLN, sentinel lymph node; LNI, lymph node invasion

**Table 7:** Table to calculate the predictive accuracy of detecting positive lymph nodes based on the updated nomogram of Winter et al. (2017b) using sentinel lymph node dissection as a reference standard.

Nomogram- based risk group (%)	Histologically positive SLNs; "right-positive" (n = 136)	Histologically positive Non-SLNs; "false-negative" (n = 128)	Total (n = 264)
5.01–10	4	0	4
10.01–15	2	0	2
15.01–20	8	1	9
20.01–25	6	2	8
25.01–30	6	12	18
30.01–35	4	1	5
35.01–40	2	1	3
40.01–45	13	10	23
45.01–50	9	15	24
50.01–55	0	5	5
55.01–60	12	4	16
60.01–65	9	3	12
65.01–70	4	6	10
70.01–75	18	3	21
75.01–80	2	7	9
80.01–85	20	18	38
85.01–90	9	8	17
90.01–95	3	2	5
95.01–100	5	30	35

The nomogram-based risk groups were calculated by applying the updated nomogram of Winter et al. ( 2017b). sLND, sentinel lymph node dissectio;.SLN, sentinel lymph node; LNI, lymph node invasion.

**Table 8:** Table to calculate the predictive accuracy of detecting positive lymph nodes based on the updated nomogram of Winter et al. (2017b) using extended lymph node dissection as a reference standard.

Nomogram-based risk group (%)	Histologically positive LNs in the eLND template; "right-positive" (n = 244)	Histologically positive LNs outside the eLND template; "false-negative" (n = 20)	Total (n = 264)
5.01–10	4	0	4
10.01–15	2	0	2
15.01–20	8	1	9
20.01–25	8	0	8
25.01–30	18	0	18
30.01–35	3	2	5
35.01–40	3	0	3
40.01–45	20	3	23
45.01–50	24	0	24
50.01–55	5	0	5
55.01–60	16	0	16
60.01–65	10	2	12
65.01–70	10	0	10
70.01–75	19	2	21
75.01–80	6	3	9
80.01–85	35	3	38
85.01–90	17	0	17
90.01–95	4	1	5
95.01–100	32	3	35

The nomogram-based risk groups were calculated by applying the updated nomogram of Winter et al. (2017b). eLND, extended lymph node dissection; SLN, sentinel lymph node.

## Discussion

To the best of our knowledge, this is the first study to examine the relationship between sentinel tracer uptake, in this case magnetic activity, and the presence of metastases in PCa. Even for radioactive marking with ^99m^Tc nanocolloid, this has not yet been investigated in PCa. Although there seemed to be no direct correlation due to a high heterogeneity in tracer uptake, this “negative” information provides important insights for clinical use. We conclude that the “10% rule,” which provided reliable results in SLN biopsy for mammary carcinoma, cannot be transferred to PCa. It must be considered that because of the often multifocal occurrence of tumors within the prostate gland, a direct peritumoral injection of the tracer with the sole representation of the primary draining tumor LNs is not possible in PCa. The tissue composition of the prostate, heterogenic tracer uptake, advanced tumor, or the presence of macrometastases with a blocked lymph pathway can also influence tracer distribution, especially in high-risk PCa (Morgan-Parkes, 1995; Weckermann et al., 2007).

However, the results suggest that the use of intraprostatically injected SPIONs combined with the intraoperative use of a handheld magnetometer is a reliable replacement/substitute for eLND in intermediate-risk PCa patients. Our data show no loss in detection of LN-positive patients, while the number of removed LNs was halved. In addition to reducing morbidity, the total histopathological effort can also be reduced so that the precision/extent of histopathological examination per node can be increased.

These results verify the conclusions of a previous study by Winter et al. (2014) that included 19 intermediate- or high-risk PCa patients and also reported 100% sensitivity for this magnetic sentinel approach, excluding one LN-positive patient who showed no SLNs following transurethral prostate resection. Winter et al. (2017a; 2018a) also reported a diagnostic rate of 100% and a sensitivity of 85.7% in a study group of 50 PCa patients and 100% diagnostic rate with a sensitivity of 96.6% in a study of 104 PCa patients. They and others had already highlighted limitations of sLND (e.g., previous hormonal treatment), so our exclusion criteria could be adjusted to improve the reliability of sLND in this study (Wawroschek et al., 2001; Weckermann et al., 2007). Nevertheless, our data and the reports of others showed that there are still limitations, especially concerning high-risk PCa. Although the number of dissected LNs could also be halved using sLND, nine LN-positive patients (7.44%) were not found. The number of missed patients was even higher than the additional diagnostic value, as only two patients with LN metastases outside the eLND template were detected (1.65%). Conspicuously, in seven of the nine false-negative patients, a maximum of two magnetically active LNs were found, and they all had a pathological tumor stage of 3b or 4. That so few SLNs could be detected in these high-risk cases may be caused by advanced PCa with high tumor burden in the prostate and blocked lymphatic vessels, which could disrupt or redirect lymphatic spread, as previously described (Morgan-Parkes, 1995). If sLND alone was only performed when a minimum of three SLNs were detected, a sensitivity of 96.43% for detecting LN-positive patients would have been reached in this cohort. Consequently, we conclude that in our cohort even high-risk PCa patients were correctly classified as LN-positive or -negative by sLND alone if there were at least three SLNs. If fewer than three SLNs were intraoperatively detected, sLND should be combined with eLND. Thus, if at least three marked SLNs are found in either intermediate- or high-risk PCa patients, the number of dissected LNs could be halved, so that sLND after intraprostatic injection of SPIONs enables a reduction in morbidity by minimizing the extent of LND while still ensuring a high diagnostic value that is comparable to eLND.

However, when examining these data with the aim of correctly detecting every positive LN, sLND seems to not be a suitable procedure for any of these risk groups, as there was a high rate of nonactive but nevertheless positive LNs. Nearly half of the positive LNs in high-risk patients did not show any magnetic activity, while 25% of the positive LNs in intermediate-risk patients were nonactive. However, fully metastasized LNs or blocked lymph pathways can redirect the tracer, as has already been described for lymphatic spread (Morgan-Parkes, 1995), and LNs not detected by sLND might already be connected downstream. Thus, if the goal is to remove as many positive LNs as possible, not only SLNs, then sLND must be combined with eLND.

This is the first study to investigate the influence of the level of magnetic activity, which reflects the extent of tracer uptake, on metastases detection for PCa. Regarding the assumption of a correlation between *in vivo* activity and LN positivity or negativity, we have to refute this hypothesis on the basis of our data. There was only a small difference between the groups, so the level of activity does not allow conclusions to be drawn. A possible explanation for a minimal higher magnetic activity of SLNs in intermediate-risk than in high-risk patients may be fully metastasized LNs or blocked lymph pathways caused by high-volume PCa, as described above, which is more likely found in high-risk PCa patients (Morgan-Parkes, 1995).

In 2000, Martin et al. (2000) were the first to introduce the “10% rule” for breast cancer using both blue dye and radioactive colloid injection in a prospective multicenter study including 758 patients. They identified a 5.8% FN rate when all nodes with 10% or more of the activity count of the hottest node were removed. Both in breast cancer and in malignant melanoma, the 10% rule was mostly seen as a good alternative to eLND (Carlson et al., 2002; Chung et al., 2008). Chung et al. (2008) found that only 1.7% of all positive nodes would have been missed when the 10% rule was performed if more than one SLN was identified. Carlson et al. (2002) had a similar FN rate, as 2.1% of positive SLNs would not have been dissected. If the extent of LND on SLNs with at least 10% magnetic activity of the most active SLN of the same patient would have been limited, one of the 13 intermediate-risk patients (7.7%) and 12 of the 65 high-risk patients (18.5%) would have been missed. It is advisable to check in particular the results of our intermediate-risk group, as our collective is too small to make a statement. Nevertheless, for both of our study groups, we cannot recommend the performance of the 10% rule, as FN rates of 7.7% or 18.5% are not acceptable. This may be caused by the enormous spread in width of the LN’s magnetic activity in both groups and the high number of nonactive positive LNs. However, we found that the FN rate could be reduced from 7.7% to 0% for intermediate-risk PCa and from 18.5% to 13.8% for high-risk PCa if all *in vivo* magnetically active SLNs are dissected without including nonactive LNs. This would still offer the opportunity to reduce the number of dissected LNs, so that in both risk groups nearly half of all LNs could be left to reduce morbidity. If this had been followed, only 812 of 1,637 LNs in intermediate-risk patients and 967 of 2,074 LNs in high-risk patients would have been dissected. If patients who had a maximum of two SLNs were additionally excluded, the FN rate for high-risk PCa could have been reduced to 3.8%. However, it should be pointed out that positive non-SLNs would then be left behind, especially in high-risk PCa.

In 2015, Winter et al. (2015b) created a nomogram for predicting the probability of LN positivity in PCa patients based on preoperative PSA, clinical T category, and biopsy Gleason score after proofing associations between univariate predictors and LNI probability. They improved their nomogram in 2017 by splitting the biopsy Gleason score into primary and secondary Gleason grades and adding the percentage of positive cores to the predicting characteristics, so that the predictive accuracy (AUC) increased from 82% to 83.5% (Winter et al., 2017b).When applying the nomogram to the cohort investigated in this study, an AUC of 81.7% showed a similarly high accuracy for predicting LNI that was comparable to other nomograms based on conventional pelvic LND (Cagiannos et al., 2003; Briganti et al., 2006b). As no positive LNs were found in any patient with a risk of LNI ≤5%, the nomogram leads to the assumption that lymphadenectomy can be dispensed with in this low risk range. This threshold of at least 5% is also recommended for performing eLND in the EAU Guidelines for PCa (Mottet et al., 2017). After applying an eLND-based nomogram, Briganti et al. (2012) also declared a cutoff value of 5% to be appropriate, as two-thirds of the patients (65.5%) could be spared eLND by missing only 1.5% with LNI (sensitivity of 87.8%, specificity of 70.3%, NPV of 98.4%).

In the risk range between 5.01% and 45%, all 27 positive patients were correctly identified when performing sLND; thus, eLND did not provide any added value in this risk group for patients in this cohort. sLND would have been sufficient to reduce morbidity in the 130 patients (60%) classified in this risk group, so that the number of dissected LNs could have been halved. In the risk range from 45.01% to 100%, both sLND and eLND showed additional diagnostic benefit, so we recommend a combination of these two to increase diagnostic reliability.

With the aim of removing all pelvic LN metastases to achieve possible therapeutic benefits, patients with a risk of ≤5% could have been spared eLND, as no positive LNs were found in this risk group. In patients with an LNI risk between 5.01% and 20% (36% of all patients), a sole sLND would have been sufficient to dissect almost all positive LNs. We recommend combined sLND with subsequent eLND for all patients with a risk between 20.01% and 100% because neither LND method alone could detect all positive LNs. Moreover, more dissected LNs seemed to increase the likelihood of finding LNI (Cagiannos et al., 2003; Heidenreich et al., 2002; Briganti et al., 2006b).

Nevertheless, we must also note that the reduced perioperative morbidity by avoiding LND in patients with a risk of LNI ≤5% must be weighed against the improved survival by removing LN metastases. Especially in patients with minimal LNI, LND can also play a therapeutic role in addition to its diagnostic benefit (Briganti et al., 2009b; Seiler et al., 2014).

Furthermore, in clinical routine, the use of nomograms is associated with an increased preoperative effort. In addition to the respective calculation, the resulting individualized counseling of patients is more complex. However, electronic nomogram tools as the online available nomogram developed by us (https://www.prostatakarzinomzentrum.info/vorhersagetool_fuer_lymphknotenmetastasen__nomogramm_.html) can provide support in this process.

The limitations of this study are the relatively small sample size, which due to unicentric data collection may not be representative of other cohorts. Although our results are comparable to previous studies, we recommend external validation and renewed data collection with a larger cohort.

## Conclusion

sLND after intraprostatic SPION injection is a reliable alternative to eLND in intermediate-risk PCa patients to correctly identify all positive patients while halving the number of dissected LNs. In high-risk PCa patients, sLND alone can also ensure the diagnostic standard if it is performed only under the condition that at least three *in vivo* SLNs are detected. However, especially in high-risk PCa, positive non-SLNs will then be left behind. So if the aim is to find all positive LNs or if fewer than three SLNs can be found, sLND should be combined with eLND. From the level of magnetic activity, no clear conclusions can be drawn concerning the presence of metastases. Use of the “10% rule” is not recommended, as an unacceptably large number of positive patients would be missed. If a risk stratification is made based on an LNI predicting nomogram, in accordance with the EAU Guidelines for PCa, it can be dispensed with for an (s)LND at a risk range for LNI up to 5%. Between a nomogram risk from 5.01% to 20%, sLND alone might be sufficient. However, external validation and renewed data collection with a larger number of cases are required.

## Data Availability Statement

The datasets generated for this study are available on request to the corresponding author.

## Ethics Statement

The studies involving human participants were reviewed and approved by Medical Ethics Committee of the Carl von Ossietzky University Oldenburg. The patients/participants provided their written informed consent to participate in this study.

## Author Contributions

AW, SE, and FW conceived and designed the study. AW and FW performed a large number of the surgeries. WG evaluated data and wrote the manuscript. SE and PA performed and documented magnetic measurements. FW, HG, and JS conducted critical revision for important intellectual content. All authors helped prepare the paper and approved the final version.

## Conflict of Interest

The authors declare that the research was conducted in the absence of any commercial or financial relationships that could be construed as a potential conflict of interest.

## Abbreviations

AUC, area under the curve; eLND, extended lymph node dissection; FN, false-negative; FP, false-positive; GS, Gleason score; LN, lymph node; LNI, lymph node invasion; LND, lymph node dissection; MRI, magnetic resonance imaging; NPV, negative predictive value; PCa, prostate cancer; PPV, positive predictive value; PSA, prostate-specific antigen; ROC, receiver operating characteristic; RP, radical prostatectomy; SLN, sentinel lymph node; sLND, sentinel lymph node dissection; SPION, superparamagnetic iron oxide nanoparticles.
